# The Alleviating Effect of Taxifolin on Deoxynivalenol-Induced Damage in Porcine Intestinal Epithelial Cells

**DOI:** 10.3390/vetsci11040156

**Published:** 2024-03-30

**Authors:** Min Zhu, Yongxia Fang, Yujie Cheng, E Xu, Yiyu Zhang, Zhenya Zhai

**Affiliations:** 1 Key Laboratory of Animal Genetics, Breeding and Reproduction in the Plateau Mountainous Region, Ministry of Education, College of Animal Science, Guizhou University, Guiyang 550025, China; 18708634765@163.com (Y.F.); 18285695928@163.com (Y.C.); exu@gzu.edu.cn (E.X.); yyzhang1@gzu.edu.cn (Y.Z.); 2 Institute of Animal Nutrition and Feed Science, Guizhou University, Guiyang 550025, China; 3 Jiangxi Functional Feed Additive Engineering Laboratory, Institute of Biological Resource, Jiangxi Academy of Sciences, Nanchang 330096, China

**Keywords:** taxifolin, deoxynivalenol, IPEC-J2 cells, Nrf2 signaling pathway, antioxidation

## Abstract

**Simple Summary:**

Deoxynivalenol (DON), a common mycotoxin widely found in feed, often causes intestinal oxidative damage, and the utilization of antioxidants is one way to reduce this damage. Taxifolin (TA) is a flavanone with tremendous antioxidant capacity. The aim of the current study was to explore whether TA alleviates DON-induced damage in porcine intestinal epithelial cells (IPEC-J2) and, if so, the underlying mechanism. Our results demonstrated that TA attenuated DON-induced cellular damage via suppressing the DON-induced declines in IPEC-J2 cell viability and proliferation, cellular apoptosis, and reactive oxygen species (ROS) production; the decline in cellular barrier function; and the decrease in antioxidant status. Collectively, our findings suggest that the damage caused by DON in the IPEC-J2 cells could be alleviated by TA, potentially through the activation of the Nrf2 signaling pathway.

**Abstract:**

Deoxynivalenol (DON) contamination in feed is a global concern that severely threatens the health of animals and humans. Taxifolin (TA) is a natural flavonoid, a member of the polyphenols, that possesses robust antioxidant properties. This study aimed to investigate the effect of TA on DON-induced damage in porcine intestinal epithelial cells (IPEC-J2). The cells were pre-incubated with a series of concentrations of TA for 24 h and exposed to DON (0.5 μg/mL) for another 24 h. The results showed that pretreatment with TA (150 μM) significantly inhibited the DON-induced decline in cell viability (*p* < 0.05) and cell proliferation (*p* < 0.01). Additionally, 150 μM TA also alleviated DON-induced apoptosis (*p* < 0.01). Moreover, TA decreased the production of reactive oxygen species (ROS) induced by DON (*p* < 0.01). In addition, TA attenuated DON-induced cell junction damage (*p* < 0.05). Further experiments showed that TA reversed the DON-induced reduction in antioxidant capacity in the IPEC-J2 cells, probably via activating the Nrf2 signaling pathway (*p* < 0.05). Collectively, these findings suggest that 150 μM TA can protect against 0.5 μg/mL DON-induced damage to IPEC-J2 cells, potentially via the activation of the Nrf2 signaling pathway. This study provides insight into TA’s potential to act as a green feed additive in the pig farming industry and its efficacy in counteracting DON-induced intestinal damage.

## 1. Introduction

A common problem in modern pig production is feedstuffs becoming contaminated by mycotoxins, especially deoxynivalenol (DON). DON is a high-toxicity fungal secondary metabolite that contaminates food and feed worldwide [1]. Pigs fed DON-contaminated feed will present anorexia, vomiting, diarrhea, growth performance decline, and other clinical symptoms [2]. DON in animal feed can contaminate animal by-products such as meat, eggs, and milk, meaning that it can easily enter the food chain and pose a threat to human health and safety [3]. Pigs are more sensitive to DON than poultry, ruminants, rats, or mice [4].

Gut health is one of the crucial factors that needs to be considered to ensure production efficiency and animal welfare. The small intestine is responsible for digesting and absorbing nutrients, as well as protecting against external stimuli such as microorganisms, harmful antigens, and toxins (including DON) [5]. Given that DON usually enters the body orally, the intestine has been validated as the major target of feed-borne DON [2]. DON exposure can damage the intestinal mucosa, increase its permeability, trigger apoptosis, and reduce antioxidant function [6]. However, at present, there is no specific antidote for DON poisoning [7]. Additionally, the mechanism underlying the toxicity caused by DON remains unclear. Elucidating the potential mechanism underlying DON-triggered toxicity may contribute to the development of protective strategies for attenuating it and help maintain intestinal homeostasis and health, thus benefitting the pig farming industry.

Recent studies have demonstrated that polyphenols can effectively alleviate the damage caused by DON [8,9]. Taxifolin (TA), a polyphenolic substance also known as dihydroquercetin (DHQ), is a dihydroflavonol compound that belongs to the vitamin P family. It has been demonstrated to have various physiological functions and pharmacological activities, such as antioxidant, anti-inflammatory, and anticancer biological activities [10]. It has also been established as a safe food supplement through evaluations of its toxicity [11] and the enhancement of its antioxidant capacity to improve production performance [12]. TA has many promising health-promoting effects. However, reports on promoting intestinal health and alleviating DON-induced damage in pigs are rare, and whether or not TA can alleviate DON-induced damage is still unknown, as is the mechanism that underlies this possible alleviative effect.

As pigs are economically important animals in animal husbandry and the agricultural sector, their intestinal health has always been a concern. The IPEC-J2 cell line is a well-established in vitro model used to study the intestinal physiology of pigs. In this study, the IPEC-J2 cell line was used as an in vitro tool to investigate whether TA plays a protective role in DON exposure-induced cellular damage and, if so, further elucidate the molecular mechanism underlying its protective effect.

## 2. Materials and Methods

### 2.1. Chemicals and Reagents

TA (CAS No.: 480-18-2, purity 99.97%, HY-N0136) and DON (CAS No.: 51481-10-8, purity 99.397%, HY-N6684) were purchased from MCE (Monmouth Junction, NJ, USA). DMEM medium was procured from Gibco (Waltham, MA, USA), and fetal bovine serum (FBS) was procured from XP BioMed (VivaCell, Shanghai, China). CCK-8 reagent was obtained from APExBIO (K1018, Houston, TX, USA). The RNA Quick Purification kit (RN001) we used was purchased from ES science (Shanghai, China). RT-qPCR SYBR Green Master Mix (11202ES08) was purchased from Yeasen Biotechnology (Shanghai, China). Antibody Ki67 (NB500-170) was procured from Novus (Littleton, CO, USA). Occludin (#91131) was procured from CST (Danvers, MA, USA). Nrf2 (380773) was procured from Zenbio (Chengdu, China). NQO1 (11451-1-AP) was procured from Proteintech (Wuhan, China). Goat antirabbit IgG (abs20002) was procured from Absin (Shanghai, China). Cy3-conjugated goat antirabbit IgG (PMK-014-096M) was procured from Bioprimacy (Wuhan, China). ECL reagent (P10200) was obtained from NCM Biotechnology (Suzhou, China). The ROS Assay Kit (S0033S) and TUNEL kit (C1086) were purchased from Beyotime (Shanghai, China). BCA protein kits (23225) were obtained from Thermo Fisher (Waltham, MA, USA).

### 2.2. Cell Culture and Treatment

The IPEC-J2 cell line is an established cell line that was generously provided to us by the academician Yulong Yin and his team at the Institute of Subtropical Agriculture, Chinese Academy of Sciences, Changsha, China. The cells were cultured in DMEM medium containing 10% FBS and 1% Penicillin–Streptomycin–Amphotericin B Solution in an incubator filled with 5% CO_2_ and at 37 °C. The cells were incubated overnight in the incubator before receiving further treatment.

For the TA experiment, the cells were treated with 50, 100, 150, 200, 300, and 400 μM TA for 24 h (for concentration screening). The concentration of TA was chosen based on the work published in reference [13], with some modifications. For the TA + DON experiment, the cells were first pre-incubated with 50, 100, 150, 200, 300, and 400 μM TA for 24 h. After that, the medium containing TA was removed, and 0.5 μg/mL DON was added to the cells for another 24 h. We chose to use a 0.5 µg/mL concentration of DON based on a previously published study and our previous study [6,14].

### 2.3. Cell Viability Detection

At the end of the processing time point, the culture medium was fully removed, and 10 μL CCK-8 reagent dissolved in a complete medium with a ratio of 1:10 was added to each well. After a 2 h incubation period, absorbance was measured at 450 nm using a Bio-Tek microplate reader (Winooski, VT, USA).

### 2.4. Cell Proliferation Assay

Ki67 staining was used to evaluate cell proliferation. The cells treated with TA (100 μM and 150 μM) or DON after TA pretreatment were fixed, and the cells were incubated with Ki67 antibody overnight. After staining with a fluorescent secondary antibody for 2 h, images were captured using a fluorescence microscope (Nikon, Tokyo, Japan). Proliferating cells showed red fluorescence, and nuclei showed blue fluorescence. The number of Ki67-positive cells (red nuclei) was calculated as a percentage of the total number of cells (blue nuclei) to assess cell proliferation.

### 2.5. TUNEL Staining

TUNEL staining was performed according to the manufacturer’s instructions (Beyotime). In brief, the cells were observed after fixation, permeability, staining, PBS cleaning, and other steps. The FITC-labeled TUNEL-positive cells were imaged using fluorescent microscopy with 488 nm excitation and 525 nm emission.

### 2.6. Measurement of ROS Production

The ROS Assay Kit (Beyotime, S0033) was applied to determine intracellular ROS. IPEC-J2 cells were inoculated in six-well plates. The cells were treated with or without TA and DON. At the end of the treatment, the cells were digested with trypsin enzyme, incubated with 10 μM DCFH-DA in the dark for 60 min, and then detected using a fluorescence microplate (Cytation5, BioTek, Winooski, VT, USA). The fluorescence intensity is expressed as the measurement obtained when excited at 488 nm and emitted at 525 nm.

### 2.7. Quantitative Real-Time PCR

IPEC-J2 cells were placed in 6-well plates at a density of 3×10^5^/well. After the cells reached 40–50% confluency, they were incubated with TA or DON treatment. RNA was extracted by adhering to the instructions provided by the manufacturer. The obtained total RNA was then transcribed into cDNA at a dose of 1 μg. Quantitative real-time PCR (RT-qPCR) was conducted using the obtained cDNA and certified procedure, and the primers are shown in Table 1. The RT-qPCR data were analyzed using the 2^−ΔΔCt^ method.

### 2.8. Western Blotting

The cells were broken down using a lysis buffer. The protein concentration of each group was measured using a BCA kit, and the concentration of each group was adjusted to make it consistent. The cellular protein was then denatured. Next, the denatured protein was separated using SDS-PAGE and transferred to a PVDF membrane. The PVDF membrane was blocked using skimmed milk powder and left overnight with the primary antibody. Afterward, the membrane was incubated with the corresponding secondary antibody, and finally, the protein band was visualized using an ECL reagent. Lastly, the gray value of each band was analyzed using Image J software (Version 1.8.0 112, National Institutes of Health, Bethesda, MD, USA).

### 2.9. Statistical Analysis

Data were analyzed utilizing SPSS 22.0 software (SPSS Inc., Chicago, IL, USA). In this paper, the results are displayed in the form of mean ± standard error of the mean (SEM). A *t*-test was used to analyze data between two groups, and a one-way ANOVA followed by Tukey’s post hoc test was employed for the analysis of data in three or more groups. A *p*-value of less than 0.05 indicated that the difference was statistically significant.

## 3. Results

### 3.1. TA Attenuated the Reduction in Cell Viability and Proliferation Caused by DON

**Figure 1:**
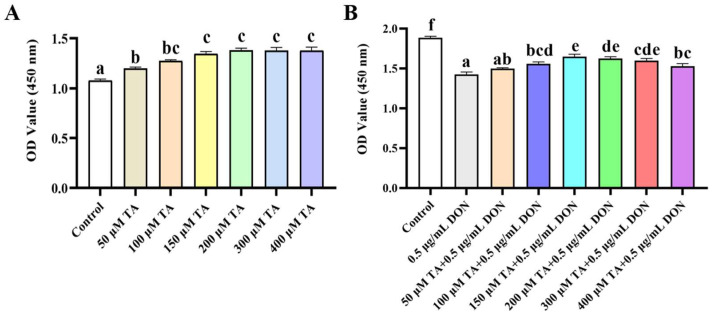
The effect of TA on the viability of IPEC-J2 cells exposed to DON. (**A**) IPEC-J2 cells were treated with TA (0, 50, 100, 150, 300, and 400 μM) for 24 h. Cell viability was determined using the CCK-8 assay. (**B**) After pretreatment with different concentrations of TA (50, 100, 150, 200, 300, and 400 μM) for 24 h, the cells were exposed to DON (0.5 μg/mL) for another 24 h. Data are shown as mean ± SEM (*n* = 6). Significant differences are indicated by different letters; the same set of letters indicates no significant difference between groups.

The effect of TA on the viability of the IPEC-J2 cells was evaluated using the CCK-8 assay. As depicted in Figure 1A, 50, 100, 150, 200, 300, and 400 μM TA significantly increased the viability of the IPEC-J2 cells (*p* < 0.05). The 150 μM concentration exhibited a significantly better effect than the 50 μM concentration (*p* < 0.05), while there was no significant difference among the 100, 200, 300, and 400 μM concentrations (*p* > 0.05). However, there is a tendency of difference between 100 μM TA and 150 μM TA concentrations (*p* = 0.053). Therefore, the 150 μM concentration was chosen for further experiments. The results also indicate that 50–400 μM TA significantly increased cell viability without any toxicity (*p* < 0.05).

To assess whether TA could alleviate the toxic damage caused by DON, the effect of TA on cell viability under DON-induced damage was determined. The results showed that the decrease in cell viability induced by DON was significantly alleviated by 100, 150, 200, 300, and 400 μM TA (Figure 1B, *p* < 0.05). Moreover, the attenuation effect of 150 μM TA was significantly better than that of 100 and 400 μM TA (*p* < 0.05), and there was no significant difference between 150 μM, 200 μM, and 300 μM TA (*p* > 0.05).

**Figure 2:**
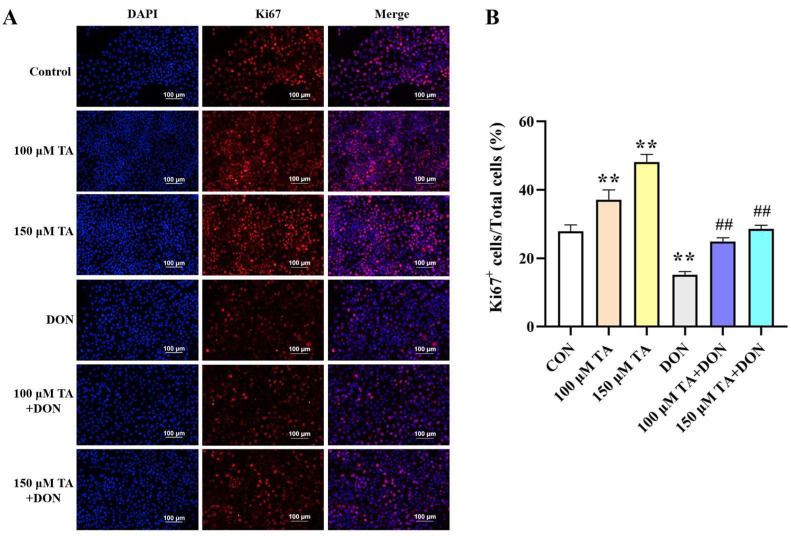
The effect of TA on the proliferation of IPEC-J2 cells exposed to DON. (**A**) Representative immunofluorescence images of the control, TA, DON, and TA + DON groups labeled with DAPI (blue) and anti-Ki67 antibody (red). Scale bar: 100 μm. (**B**) The statistical result related to (**A**). Cell proliferation was calculated as the percentage of Ki67-positive (red nuclei) cells out of all cells (blue nuclei). Data are presented as mean ± SEM (*n* = 3). Control: control group; 100 μM TA: 100 μM TA group; 150 μM TA: 150 μM TA group; DON: 0.5 μg/mL DON group; 100 μM TA + DON: 100 μM TA + 0.5 μg/mL DON group; 150 μM TA + DON: 150 μM TA + 0.5 μg/mL DON group. ** *p* < 0.01; ## *p* < 0.01. ** stands for comparison with the control group, and ## stands for comparison with the DON group.

Ki67 staining was utilized to evaluate the effect of TA on DON-induced cell proliferation impairment. Cells with red-stained nuclei were considered as Ki67-positive cells (proliferating cells). The results indicated that 100 and 150 μM TA could reverse the proliferation impairment caused by DON (Figure 2, *p* < 0.01). Based on the fact that treatment with TA can help to reduce the negative impact of DON on cell viability and proliferation, 150 μM TA has a better effect (*p* < 0.05). Therefore, 150 μM TA was selected to evaluate TA’s relieving effect on DON-induced damage in the following experiment.

### 3.2. TA Alleviated DON-Induced Apoptosis

**Figure 3:**
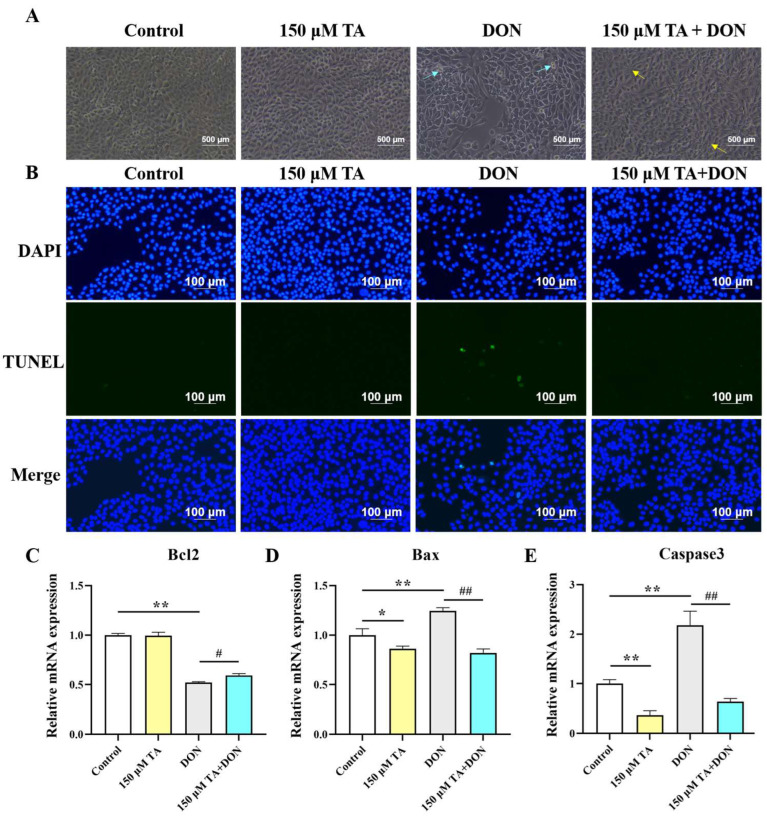
Effect of TA on DON-induced cell morphology and apoptosis. (**A**) After pretreating the cells with 150 μM TA for 24 h, they were exposed to 0.5 μg/mL DON for another 24 h. Then, cell morphology was evaluated by capturing images of the cells. The cyan arrows indicate shrunken or fragmentated cells; the yellow arrows indicate normal or dividing cells. The magnification is 40×. (**B**) TUNEL staining. (**C**–**E**) Relative expression of Bcl2, Bax, and Caspase3. Data are shown as mean ± SEM (*n* = 3). Control: control group; 150 μM TA: 150 μM TA group; DON: 0.5 μg/mL DON group; 150 μM TA + DON: 150 μM TA + 0.5 μg/mL DON group. * *p* < 0.05, ** *p* < 0.01, # *p* < 0.05, and ## *p* < 0.01. * stands for comparison with the control group, and # stands for comparison with the DON group.

Microscopy and RT-qPCR were used to observe cell morphology and detect the expression of apoptotic genes, respectively. As shown in Figure 3A, the DON treatment led to cell fragmentation and shrinkage, while the pretreatment with TA followed by the DON treatment significantly mitigated the aforementioned phenomenon. The TUNEL staining results showed that TA decreased apoptosis triggered by DON (Figure 3B). Compared with the control group, DON distinctly increased the gene expression of Caspase3 and Bax, and inhibited the Bcl2 gene (*p* < 0.01). Our findings suggest that TA did not have a significant impact on the expression of Bcl2 (Figure 3C, *p* > 0.05). However, TA was found to decrease the expression of Bax and Caspase3 in the IPEC-J2 cells (Figure 3D–E, *p* < 0.05). Accordingly, TA decreased the mRNA expression of Caspase3 and Bax (*p* < 0.01) and promoted the expression of the Bcl2 gene (*p* < 0.05) in the TA + DON group compared with the DON group (Figure 3C–E).

### 3.3. TA Decreased DON-Induced ROS Production

**Figure 4:**
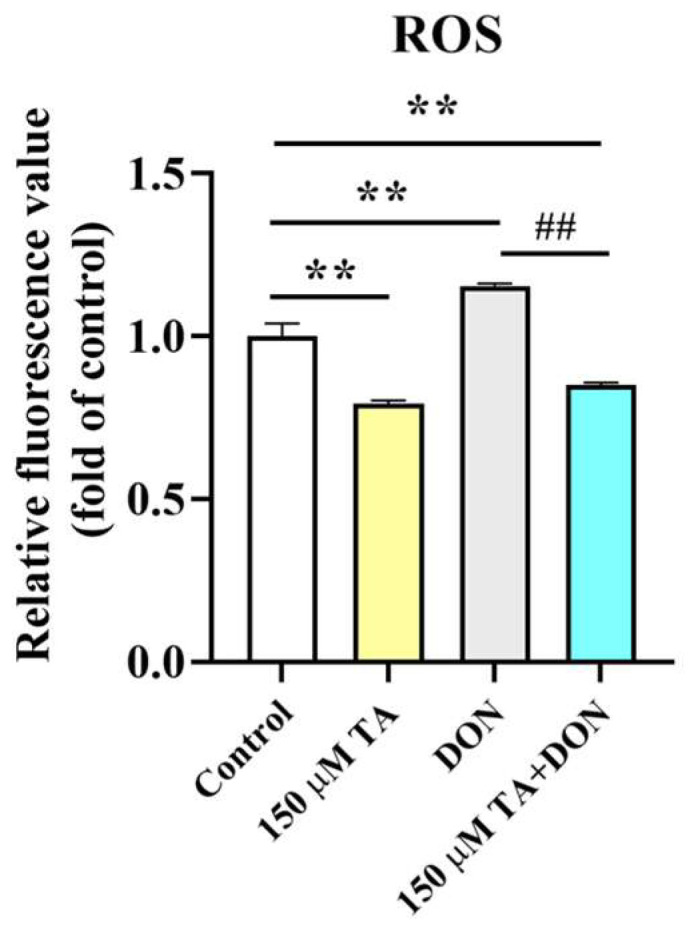
Effect of TA on intracellular ROS levels. Fluorescence values were determined using a fluorescence microplate. Data are shown as mean ± SEM (*n* = 3). Control: control group; 150 μM TA: 150 μM TA group; DON: 0.5 μg/mL DON group; 150 μM TA + DON: 150 μM TA + 0.5 μg/mL DON group. ** *p*< 0.01; ## *p* < 0.01. ** stands for comparison with the control group, and ## stands for comparison with the DON group.

After DON was applied in conjunction with TA to treat the IPEC-J2 cells, compared with the control group, an increase in ROS accumulation was observed in the DON group (*p* < 0.01). However, a reduction in ROS production compared with the control group (*p* < 0.01) was observed after TA treatment. Moreover, compared to the DON group, the TA + DON treatment led to a decrease in intracellular ROS concentration (Figure 4, *p* < 0.05).

### 3.4. TA Attenuated DON-Induced Cellular Tight Junction Damage

**Figure 5:**
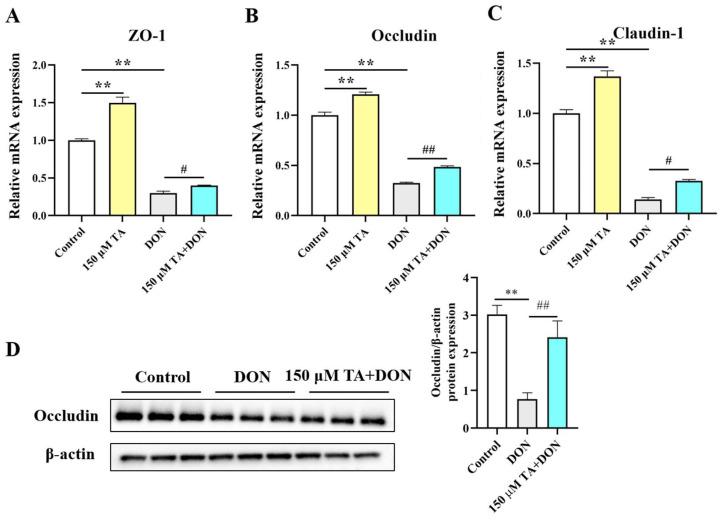
Effect of TA on intestinal tight junction protein expression. (**A**–**C**) ZO-1, occludin, and Claudin1 mRNA expressions in IPEC-J2 cells. (**D**) Occludin expression. The data are shown as mean ± SEM (*n* = 3). Control: control group; 150 μM TA: 150 μM TA group; DON: 0.5 μg/mL DON group; 150 μM TA + DON: 150 μM TA + 0.5 μg/mL DON group. ** *p*< 0.01, # *p* < 0.05, and ## *p* < 0.01. ** stands for comparison with the control group, and # stands for comparison with the DON group.

The intestinal physical barrier is crucial for gut health. Its integrity depends on the tightness of the connections in the intestines. When harmful substances or pathogens damage the gut, its integrity is disrupted. We observed that DON significantly inhibited the mRNA expressions of ZO-1, occludin, and Claudin1 (*p* < 0.01). TA pretreatment reversed the DON-induced inhibition of the mRNA expressions of ZO-1 (*p* < 0.05), occludin (*p* < 0.01), and Claudin1 (*p* < 0.05) (Figure 5A–C). The expression of the protein occludin was also determined. The Western blotting results are in agreement with the RT-qPCR results. Compared with the DON group, occludin expression was increased in the TA + DON group (Figure 5D, *p* < 0.05). Additionally, the group of cells treated with TA alone showed a significantly up-regulated tight junction expression (Figure 5, *p* < 0.01).

### 3.5. TA Attenuated the DON-Induced Decline in Antioxidant Ability in IPEC-J2 Cells

**Figure 6:**
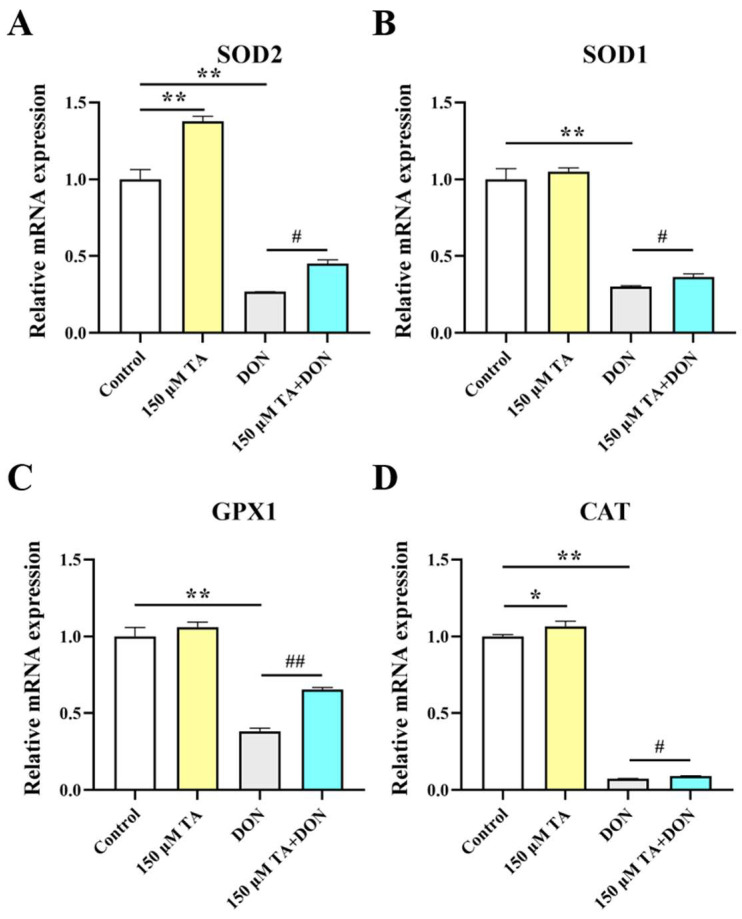
The effect of TA on the antioxidant capacity of IPEC-J2 cells under DON exposure. (**A**–**D**) The relative expression of SOD2, SOD1, GPX1, and CAT. Data are shown as mean ± SEM (*n* = 3). Control: control group; 150 μM TA: 150 μM TA group; DON: 0.5 μg/mL DON group; 150 μM TA + DON: 150 μM TA + 0.5 μg/mL DON group. * *p* < 0.05; ** *p* < 0.01. # *p* < 0.05; ## *p* < 0.01. * stands for comparison with the control group, and # stands for comparison with the DON group.

Since there is a close relationship between gut health and intestinal antioxidant activity, and multiple studies have shown that polyphenols can enhance the expression of antioxidant genes [9], we decided to investigate whether TA affects intestinal antioxidant function. Our findings indicate that TA effectively increases the expression of the antioxidant enzymes SOD2 (*p* < 0.01) and CAT (*p* < 0.05) (Figure 6A,D) but does not have a significant effect on the expression of SOD1 and GPX1 (Figure 6B,C, *p* > 0.05).

Subsequently, we further investigated the changes in the antioxidant capacity of the IPEC-J2 cells under DON exposure and the effect of TA pretreatment. The results indicated that the mRNA expression of the SOD2, SOD1, GPX1, and CAT genes was significantly inhibited by DON (*p* < 0.01), and pretreatment with TA significantly attenuated the DON-induced decreases in the mRNA expression levels of the SOD2 (*p* < 0.05), SOD1 (*p* < 0.05), GPX1 (*p* < 0.01), and CAT (*p* < 0.05) genes (Figure 6).

### 3.6. TA Mitigated DON-Induced Cellular Damage, Potentially by Activating the Nrf2 Signaling Pathway in IPEC- J2 Cells

**Figure 7:**
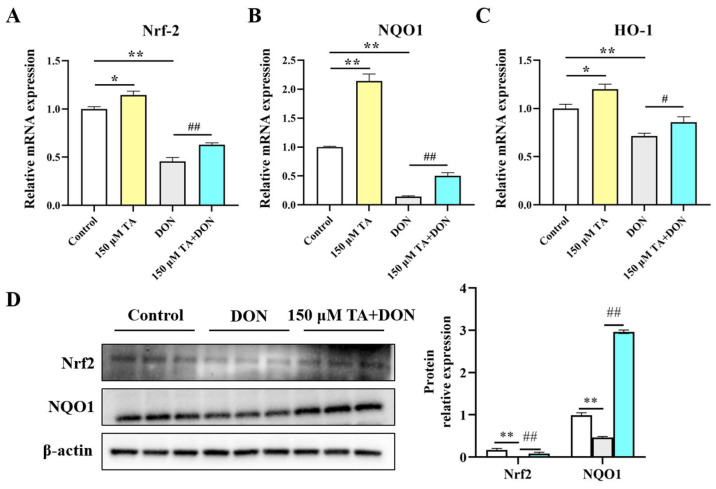
The effect of TA on the Nrf2 signaling pathway in IPEC-J2 cells under DON exposure. (**A**–**C**) The relative expression of Nrf2, NQO1, and HO-1. (**D**) Nrf2 and NQO1 expression. Data are shown as mean ± SEM (*n* = 3). Control: control group; 150 μM TA: 150 μM TA group; DON: 0.5 μg/mL DON group; 150 μM TA + DON: 150 μM TA + 0.5 μg/mL DON group. * *p* < 0.05; ** *p* < 0.01. # *p* < 0.05; ## *p* < 0.01. * stands for comparison with the control group, and # stands for comparison with the DON group.

TA’s antioxidant ability may be attributable to the activation of the Nrf2 signaling pathway. Compared to the control group, TA significantly increased the mRNA expression levels of Nrf2 (*p* < 0.05), NQO1 (*p* < 0.01), and HO-1 (*p* < 0.05). DON inhibited the level of Nrf2 and its downstream critical targets NQO1 and HO-1 (*p* < 0.01), and importantly, TA pretreatment could weaken this phenomenon (Figure 7A–C). In comparison with the DON group, the addition of TA increased the mRNA expression of Nrf2 (*p* < 0.01), NQO1 (*p* < 0.01), and HO-1 (*p* < 0.05). In addition, Nrf2 and NQO1 protein expression were also determined. The Western blotting results are in agreement with the RT-qPCR results. Compared with the DON group, Nrf2 and NQO1 expression was increased in the TA + DON group (Figure 7D, *p* < 0.05).

## 4. Discussion

Intestinal epithelial cells play an indispensable role in regulating the integrity, function, immune system, and microenvironment of the intestinal mucosa. Their function is regularly positively associated with intestinal health. Intestinal epithelial cells are the first to be exposed to DON, meaning that they represent the primary target for alimentary intoxication [15]. TA is a natural flavonoid with robust antioxidant capacity. It is known to have multiple biological functions; for example, it can reduce inflammation and heat stress [16,17]. As expected, in this study, TA exerted an alleviating effect on DON-induced decreases in cell viability and cell proliferation.

Intestinal injury or pathological conditions usually cause apoptosis [18]. DON enters cells to induce apoptosis and triggers a series of reactions that are detrimental to bodily health [19,20]. Caspase3, Bax, and Bcl2 play a vital role in the process of apoptosis [21]. In the current study, DON treatment significantly increased the number of shrunken and fragmented cells, significantly increased the expression of the Caspase3 and Bax genes, and inhibited the expression of Bcl2. This indicates that DON induces excessive cell apoptosis, overwhelming the intestinal apoptotic system and leading to intestinal damage. Importantly, TA pretreatment significantly resisted the DON-induced changes in the expression of the Caspase3, Bax, and Bcl2 genes. TUNEL staining results coincide with these findings. Pretreatment with chlorogenic acid reduces DON-induced IPEC-J2 damage by inhibiting cytotoxicity and apoptosis [22]. Our results align with the aforementioned results. Moreover, treatment with TA alone also inhibits apoptosis. This indicates that TA can protect against intestinal damage by resisting DON-induced intestinal cell apoptosis and also implies that the apoptosis pathway may be one of the mechanisms by which TA promotes intestinal development and protects the intestinal tract from damage triggered by DON.

The occurrence of apoptosis is usually mediated by the excessive production of reactive oxygen species (ROS) by mitochondria. Excessive ROS accumulation can lead to the destruction of the inherent redox balance of cells; exacerbate oxidative response; destroy nucleic acids, proteins, and lipids in cells; increase apoptosis; and affect bodily health [23]. Moreover, excessive ROS production usually induces oxidative stress, which prompts cell dysfunction or even cell death [24]. Research has shown that DON exposure increases intracellular ROS levels and triggers cellular apoptosis in IPEC-J2 cells [25]. The present study’s findings suggest that pretreatment with TA significantly reduced DON-induced intracellular ROS production.

Intestinal barrier dysfunction is one of the main causes of intestinal diseases [26]. Disrupting the balance between selective permeability and barrier function can lead to a wide range of intestinal and systemic diseases [27,28]. The tight junction, which is the main part of the intestinal physical barrier, is a closed channel composed of a variety of proteins; it determines the paracellular permeability of the intestinal barrier and prevents the infiltration of harmful substances and foreign pathogens into the blood [29]. The structure of tight junctions is regulated by the Claudin family, occludin family, zonula occludens (ZO) family, and other proteins. These proteins dynamically drive the assembly of tight junctions through mechanical force with membrane lipids to ensure the stability of tight junction structures [30]. Bioactive substances maintain the integrity of tight junctions to promote body health. In our study, we found that TA maintained intestinal integrity by significantly increasing the expression levels of Claudin1, occludin, and ZO-1 in IPEC-J2 cells. The destruction of the intestinal epithelial barrier leads to increased intestinal permeability, which can allow for the infiltration of pathogens, toxins, and antigens. This, in turn, can negatively impact the absorption of nutrients [31]. In this study, we also found that DON greatly decreased the expression levels of Claudin1, occludin, and ZO-1. Previous studies have also shown that DON impairs intestinal integrity and affects intestinal permeability by down-regulating the expression of tight junction proteins such as ZO, Claudins, and occludin [32,33]. Quercetin dose-dependently protects cells against DON-induced barrier dysfunction [34]. In a mouse model of diquat-induced oxidative stress, pretreatment with tannic acid improved intestinal barrier integrity by increasing the mRNA expression of the ZO [35]. Intestinal probiotics reduce intestinal permeability by improving the expression of ZO-1, occludin, and Claudin, maintaining the integrity of the intestinal barrier, and thereby improving the intestinal epithelial barrier’s functioning and preventing intestinal damage caused by pathogens and harmful substances [36]. These findings support the finding of our study that TA maintains the integrity of the intestinal barrier by enhancing the expression of intestinal tight junction proteins and preventing DON-induced intestinal barrier damage.

Intestinal damage is usually associated with oxidative stress. For example, DON-induced damage to animal bodies and cells usually causes intestinal toxicity and intestinal barrier damage by destroying the oxidation system [37]. Enhancing the expression of intestinal antioxidant markers is beneficial for promoting intestinal and body health [38]. When the body is in a state of oxidative stress, the antioxidant markers superoxide dismutase (SOD), glutathione peroxidase (GPX), and catalase (CAT) can maintain the homeostasis of the internal environment by scavenging excess free radicals in the body to prevent damage induced by free radicals causing harm to the body. Resveratrol can enhance the activity of antioxidant enzymes such as CAT, SOD, and GPX, thereby improving the antioxidant defense ability of pancreatic tissue [39]. In a previous study, quercetin increased the levels of T-AOC and CAT and the glutathione/oxidized glutathione ratio in the jejunum of weaned piglets, which was correlated with a reduction in oxidative stress and reduced diarrhea and weaning-related intestinal damage in the weaned piglets [40]. The above studies show that polyphenols help to protect the body from oxidative damage by improving antioxidant function. In our study, TA increased the expression of the main antioxidant enzymes, SOD1 and CAT. It did not have a significant effect on SOD2 and GPX1 but did have a tendency to increase their expression. The DON-induced inhibition of the expression of SOD1, SOD2, CAT, and GPX1 was significantly alleviated by TA pretreatment. This indicates that TA can improve intestinal health and prevent DON damage by enhancing intestinal antioxidant capacity. In addition, nuclear factor erythroid 2-related factor 2 (Nrf2), as a transcription factor, plays a crucial role in regulating redox balance. Many natural antioxidants have been known to reduce mycotoxin-induced oxidative stress by restoring the expression of Nrf2 and its downstream targets. Baicalin Zinc can enhance the expression levels of antioxidant genes such as HO-1 and NQO1, thereby reducing oxidative stress and increasing nutrient absorption in pigs to protect against DON-triggered damage [41]. Resveratrol can effectively alleviate DON-induced intestinal damage by enhancing intestinal antioxidant capacity and activating the Nrf2 signaling pathway [8,9]. TA increases antioxidant capacity by up-regulating the expression levels of Nrf2-related antioxidant genes such as HO-1 and NQO1 [42,43]. In this study, TA significantly increased the expression levels of Nrf2, NQO1, and HO-1. Moreover, TA reversed the DON-induced inhibition of Nrf2, NQO1, and HO-1 expression. This finding reveals that TA may be able to boost intestinal development and alleviate DON-induced intestinal damage by enhancing antioxidant capacity, potentially doing so via the Nrf2 signaling pathway.

## 5. Conclusions

In summary, 100–400 μM TA alleviated the DON (0.5 μg/mL)-induced decline in cell viability and cell proliferation, with 150 μM being established as the optimal concentration. TA also eased DON-induced cellular apoptosis and reactive oxygen species (ROS) production. Furthermore, TA attenuated a DON-induced reduction in cellular barrier function. Additionally, TA activated the Nrf2 signaling pathway and improved antioxidant properties, which were all hindered by DON. These findings were evidenced by the up-regulation of the expression of the Nrf2, HO-1, and NQO1 genes. Therefore, TA could further protect the intestine against DON-induced damage, thereby promoting intestinal development and health. The results of this study provide insights for future developments centered around utilizing TA as a green feed additive in the pig industry to counteract DON-induced intestinal toxicity.

## Figures and Tables

**Table 1:** Primer sequences of some genes for quantitative real-time PCR.

Genes (Accession Number)	Forward Primer (5′-3′)	Reverse Primer (5′-3′)	Product Size (bp)
*Nrf2* (XM_013984303.2)	TTAGATAGTGCCCCTGGAAGC	GCTTGAATGTTTGTCTTTTG	207
*HO-1* (NM_001004027.1)	TGATGGCGTCCTTGTACCAC	GACCGGGTTCTCCTTGTTGT	71
*NQO1* (NM_001159613.1)	CATGGCGGTCAGAAAAGCAC	ATGGCATACAGGTCCGACAC	135
*SOD1* (NM_001190422.1)	AAGGCCGTGTGTGTGCTGAA	GATCACCTTCAGCCAGTCCTTT	118
*SOD2* (NM_214127.2)	GGCCTACGTGAACAACCTGA	TGATTGATGTGGCCTCCACC	126
*CAT* (NM_214301.2)	TCCAGCCAGTGACCAGATGA	CCCGGTCAAAGTGAGCCATT	182
*GPX1* *(NM_214201.1)*	CCTCAAGTACGTCCGACCAG	GTGAGCATTTGCGCCATTCA	85
*Bcl2* (NM_214285.1)	GAAACCCCTAGTGCCATCAA	GGGACGTCAGGTCACTGAAT	196
*Bax* (XM_013998624.2)	ATGATCGCAGCCGTGGACACG	ACGAAGATGGTCACCGTCTGC	296
*Caspase3* (NM_214131.1)	GGAATGGCATGTCGATCTGGT	ACTGTCCGTCTCAATCCCAC	351
*ZO-1* (XM_021098827.1)	AAAGCCCTAAGTTCAATCACAATCT	TCCTCATCTTCATCATCTTCTACAG	253
*Occludin* (NM_001163647.2)	ACTGGCGGCGAGTCCTGCGACGAGC	TATTGTATTCATCAGCAGCAGCCAT	238
*Claudin1* (NM_001244539.1)	TCAGGTCTGGCTATCTTAGTTGC	CTGGAAGGCGAAGGTTTTGG	233
*β-actin* (XM_003124280.5)	TGCGGGACATCAAGGAGAAG	AGTTGAAGGTAGTTTCGTGG	216

## Data Availability

The data supporting the findings of the present study are available from the corresponding author upon reasonable request.
